# Clinical characteristics and prognostic factors in patients with pulmonary tuberculosis as a primary cause of respiratory failure admitted to intensive care units

**DOI:** 10.1186/2197-425X-3-S1-A383

**Published:** 2015-10-01

**Authors:** S-S Han, S Kim, S-J Lee, WJ Kim, Y Hong, H-Y Lee

**Affiliations:** Department of Internal Medicine, School of Medicine, Kangwon National University, Chuncheon, Korea Republic of Korea; Seoul Medical Center, Seoul, Korea, Republic of Korea; School of Medicine, Kangwon National University, Chuncheon, Korea, Republic of Korea

## Introduction

Tuberculosis (Tb) is currently the most frequent cause of death due to an infectious disease worldwide. The cases of Tb requiring intensive care present 1-3% of all patients with TB. Hospital mortality has been reported to be 60% for patients with respiratory failure due to pulmonary TB.

## Objectives

The present study sought to analyze mortality of patients in a local university hospital, and a public hospital, to whom mechanical ventilation (MV) was applied due to respiratory failure caused by pulmonary tuberculosis (PTB), and the factors that affect the mortality of patients.

## Methods

This study retrospectively investigated patients hospitalized for application of MV between January 2011 and April 2014, in Kangwon National University Hospital, and Seoul Medical Center ICUs. Medical records of the patients were investigated in the following four areas: clinical characteristics, laboratory results, radiographic findings, and progress in the ICUs. The control group consisted of patients who were hospitalized for respiratory failure due to community-acquired pneumonia (CAP). Analysis of primary outcomes was conducted by comparing the clinical patterns including mortality in PTB patients to whom MV had been applied and CAP patients. Analysis of secondary outcomes was based on 28-day mortality of TB group and the factors that affect 28-day death.

## Results

Mortality was significantly different between the two groups: 39 of 41 patients (95.1%) died in the TB group, and 37 of 59 patients (62.7%) died in the CAP group (p < 0.001). However, APACHE II did not show a significant difference between the groups, showing the mean of 20.71 ± 6.694 (p = 0.379), and the incidence of ARDS(48%, p = 0.784) and ventilator-associated pneumonia (35%, p = 0.782) did not show a significant difference between the groups. However, the TB group showed more incidences of multiple organ failure (OR 463.3, 95% CI: 2.265-94759.7, p = 0.024) and lower body mass index (OR 0.456, 95% CI: 0.217-0.960, p = 0.039), and CRP (OR 0.605, 95% CI: 0.397-0.922, p= 0.019) than the CAP group. The TB group also showed a wider extent of lung lesion intrusions (OR 14.685, 95% CI: 1.255-171.849, p = 0.032) when scored on 'modified CT score', and higher frequency of TB-destroyed lung (OR 423.315, 95% CI: 1.3-134857.4, p = 0.040). 28-day mortality in the TB group was 75.6% (31/41), and the group showed a similar degree of APACHE II and pulmonary involvement to the death group; multivariate analysis results did not find significant prognostic factors.

## Conclusions

PTB patients who were admitted to ICU with respiratory failure requiring MV showed identical APACHE II but much higher mortality compared to the CAP group. They also showed high frequency of TB-destroyed lung and multiple organ failure, as pulmonary involvement was wider than in the CAP group.

